# “A lot of them have scary tears during childbirth…” experiences of healthcare workers who care for genitally mutilated females

**DOI:** 10.1371/journal.pone.0246130

**Published:** 2021-01-29

**Authors:** Oluchukwu Loveth Obiora, Johanna Elizabeth Maree, Nokuthula Gloria Nkosi-Mafutha

**Affiliations:** Department of Nursing Education, Faculty of Health Sciences, University of the Witwatersrand, Johannesburg, South Africa; Hackensack Meridian Health, UNITED STATES

## Abstract

Despite concerted efforts to curb Female Genital Mutilation/Cutting (FGM/C), it is still a contributor to the high morbidity and mortality rates among females in Africa. According to available literature, the experiences of healthcare workers who care for the genitally mutilated females in Nigeria have not been described, hindering efforts towards ending this procedure through evidence-based, community-led interventions. This qualitative study described the experiences of healthcare workers caring for the genitally mutilated females in South-Eastern Nigeria. In-depth interviews conducted with 17 participants resulted in two themes and five sub-themes. The participants faced major challenges in caring for these females as the complications of FGM/C resulted in situations requiring advanced skills for which they were ill-prepared. Irrespective of this complex situation, the participants believed FGM/C was an age-old cultural practice; some even supported its continuation. The solution to this problem is not simple. However, educational programmes involving all cadres of healthcare workers could assist with eradicating this practice. Also, enforcing the anti-FGMC law could enhance the eradication of this procedure.

## Introduction

Female Genital Mutilation (FGM), also referred to as Female Genital Cutting (FGC) or Female Circumcision (FC), has drawn considerable attention from many organisations and researchers worldwide [1]. Even though this harmful practice is a contributor to the high morbidity and mortality rates among females, it continues among many ethnic groups, especially in African countries such as Egypt, Ethiopia and Nigeria, as it is based on myths and religion passed from generation to generation [2, 3]. However, the practice of FGM/C is found to a lesser degree in other parts of the world [4], such as in certain ethnic groups in Central and South America [5], as well as the developed world. This is due to the rise in international migration, which has increased the number of girls and women living in the various diaspora populations, including in Europe, North America and Australia, who have undergone or may undergo this practice [4, 6].

According to the WHO [7], FGM/C refers to all procedures involving partial or total removal of the external female genitalia and/or injury to the female genital organs, whether for cultural or non-therapeutic reasons. A systematic review of 56 FGM/C revealed more than one in 10 females who had undergone some form of FGM/C, including symbolic nicking of the clitoris, experienced immediate complications, although the risks increased with Type III, which is infibulation [8, 9].

Several studies have reported long- and short-term consequences of FGM/C, which included keloids formation, painful urination, menstrual problems, obstetric fistula, perinatal risks, the risk for infections, prolonged childbirth, need for later surgeries, such as de-infibulation, and psychological problems [1]. This places a great burden on the health system and the healthcare workers because genitally mutilated females might need special care and medical attention, as the long‐term health problems are in many cases irreversible [10].

It is clear that healthcare workers have a role to play in the prevention and mitigation of the negative effects of FGM/C. Traditionally, FGM/C was performed by traditional circumcisers and elderly relatives [11–13]. However, healthcare workers are sometimes the perpetrators leading to the medicalising of the procedure [1, 12]. The involvement of healthcare workers in the practice of FGM/C was one of the early interventions put forward to reduce the harm to females [14]. However, in 2012, the United Nations, human rights organisations, civil societies and professional organisations rejected medicalisation of FGM/C and declared it to be a violation of the human rights of females [15, 16].

However, Yoder et al. [17] reported that the medicalisation of FGM/C has continued in several countries. Countries that had a significant number of FGM/C reportedly performed by healthcare practitioners were Egypt, Kenya, Nigeria, Yemen, Djibouti, Guinea, Chad, Sudan, Iraq and Ghana [18]. Medical practitioners, nurses, midwives and other cadres of trained healthcare workers have reportedly perpetuated FGM/C for reasons including economic gain, pressure from the community members and a sense of duty to adhere to community requests [12, 19, 20]. In addition, some healthcare workers believe that medicalisation is a harm reduction strategy that can avert some of the immediate risks of FGM/C [12, 21].

There is insufficient evidence that medicalisation reduces documented obstetric or other long-term complications associated with FGM/C [1, 10, 20]. However, according to the WHO (2010), there are serious risks associated with medicalisation of this practice as it may give the impression that the procedure is medically sound or at least not very harmful to girls and women’s health [22]. It can also further institutionalise the procedure, rendering it a routine medical procedure, thereby influencing families who may have decided not to put their daughters through FGM/C to now see it as one of the childbirth packages [23]. Furthermore, some healthcare workers could develop some professional or financial interest in FGM/C procedure if medicalisation is legalised [22].

The research problem for this study relates to FGM/C in Nigeria, where an estimated 21.6 million women of childbearing age have undergone the procedure [24]; this accounts for 24.5% of the practice globally [25]. In Nigeria, healthcare is provided by registered nurses, midwives and medical doctors, semi-skilled community health extension workers (CHEWs) who also have some formal training which enables them to render healthcare services, and unskilled voluntary village healthcare workers (VVHW). There are also the unskilled traditional birth attendants (TBA), who are usually revered community members, who go round the villages to assist women during childbirth. Some of the TBAs also use spaces in their houses as their clinics where they render healthcare assistance to the women who consult them. Although not certified for such procedures, CHEWs and VVHWs often perform episiotomies and vaginal suturing when delivering babies [26]. However, in most rural communities in Nigeria, the VVHW and CHEWs form a larger population of the healthcare practitioners, followed by the registered nurses and midwives who primarily run the health facilities, with the medical doctors visiting the health facilities on specific days.

Concerning Nigerian Law and FGM/C, Nigeria has a federal system of government, comprising 36 states [25]. Both the Federal and State governments are involved in the enactment of laws. This implies that although the federal government is responsible for passing general laws, the State governments must adopt and implement them in their respective states. The Violence Against Persons (Prohibition) Act, 2015 (the VAPP Act), which came into force on 25 May 2015, was the first federal law attempting to prohibit FGM/C across the whole country [2, 11]. The VAPP Act aims to eliminate gender-based violence by criminalising and setting out the punishment for acts including rape (but not spousal rape), incest, domestic violence, stalking, harmful traditional practices and FGM/C [11]. However, the law does not provide a clear definition of FGM/C; it did not expressly criminalise failure to report FGM/C that has taken place or is due to take place, neither did it address FGM/C carried out by health professionals or in a medical setting [27].

Understanding the perspectives and experiences of different role players in the communities about FGM/C is crucial for understanding the roots of the practice and directing effective preventive programmes [13]. Healthcare workers are part of the key role players in FGM/C practicing communities, as they directly interact with the females undergoing FGM/C or complaining about complications of this practice [10, 12, 13]. Given their position, they can play a major role in preventing and mitigating the harmful practice of FGM/C in different contexts. According to available literature, the experiences of healthcare workers who care for the genitally mutilated females in Nigeria have not been described, hindering efforts towards ending this procedure through evidence-based, community-led interventions [1]. Therefore, this study aimed to answer the following research question: *What are the experiences of healthcare workers caring for genitally mutilated females in South East Nigeria*? The objective of the study was to describe the experiences of healthcare workers caring for the genitally mutilated females in South-Eastern Nigeria.

## Methods

### Research design

A qualitative descriptive design [28] was used for the study. According to Sandelowski [28], qualitative description allows researchers to present a descriptive summary of events occurring in everyday life, as it is amenable to obtaining straight and largely unadorned answers to questions of special relevance to practitioners and policymakers. A qualitative descriptive design also uses naturalistic inquiry without pre-selected variables and a predetermined commitment to a specific theoretical view.

### Setting

The conducting of this study was in Ebonyi and Imo States in South-East Nigeria. These states have the highest FGM/C prevalence in Nigeria, 74.2% and 68.0% respectively [25]. The common language is Igbo and the majority of the people are farmers, producing foods such as yam and cassava in large quantities, a significant amount of the population rear livestock, some were traders, while a few were civil servants.

### Population and sampling

The population for the study was all nurses, midwives, CHEWs and VVHWs in two states of South-East Nigeria. To be included in the study, these healthcare workers must have worked in the study setting for at least one year. Purposive sampling, the sampling method of choice for qualitative descriptive work [28, 29], selected the participants after ethical clearance was obtained from the Human Research Ethics Committee, the Institutional Review Board and the Heads of Primary Health Care of the study communities. The prospective participants received an explanation and an information leaflet, whereafter they gave their informed consent. Twenty (n = 20) healthcare workers were recruited, but only 17 (n = 17) volunteered and participated in the study. The reasons for refusing to participate were busy work schedules and non-willingness to discuss FGM/C.

### Data collection and instrument

In-depth interviews collected the data. One opening question asked, “*Please tell me your experience of caring for genitally mutilated females in this community*.*”* Probes and prompting questions [30] were used to enhance an in-depth discussion. The researcher listened and allowed the probes to emanate naturally from, but not limited to, the information the interviewee provided. Also, to make the interview more natural, paraphrasing, passive and indirect probes were used to avoid limiting the flow of information from the participants. Additional interviews were conducted after the transcription of the initial 10 interviews, during which certain words were clarified to ensure meaning was not lost; thereby, enhancing the rigour and validity of the study. The first author, a registered nurse-midwife who had no professional or personal relationship with the participants, collected the data in Igbo language from November 2018 to April 2019 in private corners at the participants’ various health facilities. The interviews lasted on average 25 minutes, with general information collected first. The participants received pseudonyms to ensure confidentiality, field notes were taken and the interviews were audio-recorded with the permission of the participants. A password protected computer saved the anonymous audio-recordings.

### Data analysis

Data analysis commenced alongside data collection. The first author translated the interviews from the Igbo language into English and transcribed the interviews verbatim. The general information of the participants was entered onto a spreadsheet. Qualitative content analysis, the analysis method of choice in qualitative descriptive studies [28], was used to analyse the data. An inductive approach [31, 32] was used. First was the reading of the transcriptions with notes made in the margins. Repeating this process ensured the capturing of all the keywords. The keywords were transferred onto coding sheets and subthemes were created. Subthemes belonging together were grouped into themes with higher-order headings. The first two authors were involved in the data analyses. We used reflexivity to become aware of our prejudices resulting from our experiences, knowledge and privileged positions [33, 34] and how it might influence the findings. Finally, the Consolidated Criteria for reporting qualitative research (COREQ) checklist [35] was adhered to.

## Results

### The participants

The participants consisted of 17 healthcare workers, between the ages of 25 and 51 years; their mean age was 35.7 (SD ± 7.2). The majority (12 of 17) were married. Only a few (four of 17) were registered nurses or midwives, or both, seven were Community Health Extension Workers (CHEW), while six were Voluntary Village Health Workers (VVHW) (Table 1).

**Table 1:** General information of the participants.

Healthcare workers	Age (Years)	Marital status	Professional qualification	Years of practice in the communities	Years of residency in the communities	Support for FGM/C continuation
1	49	Married	CHEW	11	Many	No
2	40	Married	RN	2	2	No
3	25	Married	RN/RM	More than 2	25	Yes
4	51	Married	RM	7	7	Yes
5	42	Married	VVHW	10	10	Not sure
6	34	Married	VVHW	16	16	Yes
7	32	Married	VVHW	More than 1	More than 1	Yes
8	26	Not disclosed	CHEW	More than 1	More than 1	No
9	38	Married	VVHW	More than 1	More than 1	Yes
10	30	Married	CHEW	8	8	No
11	38	Married	CHEW	9	9	No
12	32	Married	CHEW	4	20	No
13	28	Not disclosed	CHEW	7	More than 4	No
14	35	Not disclosed	RN/RM	8	More than 5	No
15	32	Not disclosed	CHEW	15	Many years	No
16	35	Not disclosed	CHEW	10	Many years	No
17	40	Not disclosed	VVHW	4	Many years	No

### Themes arising from the data

Two themes and five sub-themes arose from the data. The themes were providing healthcare for circumcised females and justifying the practice of FGM/C (see Table 2).

**Table 2:** Themes and sub-themes emerging from the data.

Themes	Sub-themes
Providing healthcare to circumcised females	• Complications due to FGM/C • Challenges to quality care for circumcised females
Justifying the practice of FGM/C	• FGM/C is part of the culture • The influence of the Government’s ban on FGM/C • The continuation of FGM/C

#### Theme one: Providing healthcare for circumcised females “…A lot of them have scary tears…”

This theme consisted of two sub-themes. The sub-themes were complications due to FGM/C and challenges to providing quality care for genitally mutilated females.

*Sub-theme one*: *Complications due to FGM/C*. The participants reported that circumcised females generally have lacerations, most commonly third-degree perineal tears. According to the participants, circumcised women are also prone to intrapartum and postpartum haemorrhage and other complications, such as foetal stillbirth, infections, faecal incontinence, vesicovaginal fistula (VVF), severe pain post-partum, prolonged labour and genital keloid. The participants said:

“*…The truth is that a lot of them do have scary tears during child delivery here*.” (P_2_)

“*…Some of these women have foetal stillbirth due to narrowed vaginal walls*. *Some have stool incontinence*, *infections*, *and VVF … I have also seen a circumcised woman who later had keloid formed in her genitals*, *and thus made her vagina narrow*.” (P_1_)

“*I have assisted many women with childbirth and have noticed that circumcised women usually have tight perineum during childbirth…they do undergo much pain when they have those big tears and it is more difficult to suture those sudden tears*. *So*, *the woman will still undergo real pain after childbirth when you will be trying to suture the tears…*” (P_13_)

The participants revealed some women had undergone mutilation to such an extent that they had difficulty identifying the parts of the genitals. These women were at high risk for postpartum haemorrhage. The participants said:

“*…Sometimes they will so much cut off the clitoris that you won’t even know it was ever there or you might sometimes get confused identifying the different parts of the vulva*.” (P_13_)

“… *We saw that she had infibulation*. *We had difficulty identifying the different parts of her vulva*. *It was just holes that were left for her… she said that it was her father who circumcised her*.” (P_17_)

“*You know*, *some of these women have Type III FGM/C*. *So*, *during delivery*, *they experience PPH*.” (P_3_)

Complications resulting from FGM/C were not limited to labour, as the participants also experienced the death of baby girls after circumcision. According to the participants, the government’s warnings about FGM/C led to the procedure being conducted secretly; a situation that prevented babies and women from accessing proper health care, counseling and support.

“…*The chief midwife in my institution of training had a private clinic in her house*. *One day*, *the midwife ran to work after circumcising a baby girl in her private clinic*. *She never knew that the girl was bleeding heavily*, *and her assistants tried the much they could to stop the bleeding*, *but with no success*. *The baby was then rushed to our hospital*, *but she was already so weak and so died*.” (P_1_)

“*I have witnessed three (3) babies that bled to death after being circumcised…*” (P_5_)

“*We live in this community and if they have any problem*, *they are meant to come to us and we will help them*. *But the issue is that since the campaign against FGM/C started*, *people do it secretly without telling us*. *They don’t come to us for counselling or anything about FGM/C*. *We only see that they have circumcised their daughters when they bring them for treatment of illnesses like fever*, *diarrhoea or when they present the children for immunisation*.” (P_16_)

*Sub-theme two*: *Challenges to providing quality care for genitally mutilated females*. The participants experienced various challenges in providing high quality care to the genitally mutilated females. One challenge was the shortage of skilled staff, such as registered nurses and registered midwives. Also highlighted was the lack of basic consumables. The participants explained:

“*Yes*, *we need more skilled staff here*. *Midwives are needed in this community and the various villages in it*. *Remember I told you that some villages in this community have their clinics being manned by TBAs and VVHWs*. *The majority are manned by CHEWs…*” (P_14_)

“*…Also*, *we need to have a regular supply of consumables and other reusable equipment needed for the care of these patients*. *Many village facilities don’t have electricity and good/nearby source of water supply*. *These really affect our activities*. *They should create a link between us and the hospital in town so that when we refer women*, *they will be readily attended to in the hospitals*…” (P_14_)

The participants lacked continuous education and training, as only a few RNs or RMs were fortunate to go to the cities for training. They were also not equipped to pass the knowledge on to the other healthcare workers, explained as follows:

“*We need more training on better ways to care for these females*. *They usually pick one or two staff from this big community and they go to the city when they want to do seminars and training for staff*. *There is no way one midwife will be able to impart the knowledge gained to all the healthcare workers in all the health centres in this community*. *Sometimes too they come back without the tools for all of us to learn and practice what they have been taught*.” (P_16_)

The participants were of the opinion that the government warnings about FGM/C led to the procedure being conducted secretly, hampering the provision of quality healthcare. Due to the secrecy, there was not always a referral to the hospital, for proper management, for those who experience complications during cutting.

“*We live in this community and if they have any problem*, *they are meant to come to us and we will help them*. *But the issue is that since the campaign against FGM/C started*, *people do it secretly without telling us*. *They don’t come to us for counselling or anything about FGM/C*. *We only see that they have circumcised their daughters when they bring them for treatment of illnesses like fever*, *diarrhoea or when they present the children for immunisation*.” (P_16_)

In summary, the participants experienced various challenges in terms of caring for women who were genitally mutilated. They reported that FGM/C made the females prone to complications such as third-degree lacerations during childbirth, intra and postpartum haemorrhage and foetal stillbirth during childbirth, and that women developed vesicovaginal fistula and genital keloid. Due to these complications, the participants felt they needed advanced skills to be able to render efficient healthcare services to circumcised females.

#### Theme two: Justifying the practice of FGM/C “…an age-long tradition…”

*Sub-theme one*: *FGM/C was part of the culture*. Despite having to manage the complication that genitally mutilated women experienced, some of the participants justified it by explaining FGM/C was part of their culture and had been in existence before they were born. Most reported they had undergone circumcision just like other women in the communities. According to them, almost all the women in the villages had undergone circumcision, and FGM/C was highly esteemed. However, one of the participants believed FGM/C was rooted in ignorance.

“*…FGM/C is an age-long tradition in this community…To the best of my knowledge*, *almost all the women in this village are circumcised*. *It is a way of life that has lasted for years and people see no wrong in it…FGM/C is highly esteemed in this community*” (P_12_)

“*…It is not every girl that is circumcised that do develop complications*. *It is usually very few*. *The people that do these circumcisions for them have been on the job for many years and it is not even a hard thing to do*. *So*, *don’t see it as if every circumcised woman or girl is in trouble*. *All our mothers were circumcised*, *and they lived long and had many children*, *and we are here today*. *We also were circumcised*, *and we did not die*. *We have our daughters circumcised who are themselves married with many children too*. *So please*, *it does not mean that every circumcised girl or woman will have problem or is to be pitied*, *or that circumcision will make the girl to die*. *People get sick for several reasons and die for several reasons*. *So*, *it is with childbirth too*.” (P_17_)

“*They insist on circumcising their daughters because they are circumcised themselves… Ignorance is the reason behind FGM/C*…” (P_5_)

Most of the participants did not regard the cutting to be a major procedure, comparing FGM/C to male circumcision, and said it took less than a week for complete healing of FGM/C wound. They explained:

“*When we cut off the excess flesh*, *we get a particular herb and squeeze it so that the liquid from it drops on the wound*, *then it will stop bleeding*. *Healing time is similar to the time for the healing of male circumcision*. *It takes less than a week for healing to be complete*.*”* (P_1_)

“*The circumciser uses a sharp object… to swiftly cut off the clitoris depending on what the girl’s mother agreed with the circumciser*. *The sharp object is a type of razor blade that can be washed and reused*. *It is used for both male and female circumcision*. *After the cutting*, *they use a particular herb which is popularly known to stop bleeding from injuries…the wound gradually heals by itself*.” (P_16_)

*Sub-theme two*: *The influence of the Government’s ban on FGM/C*. Some of the participants supported a ‘new’ method of mutilation they described as safe compared to the ‘traditional cutting’ method. The adopting of this method was due to the Government’s ban on FGM/C.

“*Yes…our old healthcare workers believe in FGM/C… so when the government banned it*, *they adopted this new method…In this method*, *they apply menthol and Vaseline cream on the girl-child’s clitoris at infancy stage*. *This method reduces the shape and size of the clitoris from birth… It takes between 3 and 6 months from birth to perfect it…*” (P_3_)

“*The new method is good*. *It gets the clitoris flattened as you push and massage it with Vaseline*, *so it looks good…The new method takes about 3 months to complete and it looks good*. *The clitoris is usually soft after birth*, *so you force it to flatten before it gets strong*.” (P_6_)

In addition to the ‘new’ method, adopted after the Government’s ban of GFM/C, the participants experienced the cutting occurring in secrecy. They explained:

“*…these things are done in the inner room*, *but it is against the law for healthcare workers to do FGM/C*.” (P_2_)

“*I suppose some healthcare workers might be doing FGM/C secretly*. *Not quite long*, *a man who is not an indigene of this community but lives in our village came to me with his wife who was pregnant and in her last trimester*. *He wanted her to register in our facility so that she will give birth to her child in our facility*. *The man then inquired if we circumcise female children too after they are born and we answered him in the negative*. *He then told us that he will go to another facility where his child will be circumcised after birth whether male or female*. *I tried persuading him against FGM/C but he insisted on going to another facility and left us*. *I later saw the woman with a baby girl and truly*, *the girl was circumcised*. *So that gave me the impression that some healthcare workers might still be doing FGM/C secretly*.” (P_16_)

There was a division amongst the participants in terms of what they teach women about FGM/C. Some were not willing to tell what they taught the women, whilst those who were supported the Government’s ban. They explained:

“*Well*, *it is our chief here who is a registered nurse that use to give them those talks and teachings during antenatal classes*. *She also attends anti- FGM/C meetings organised by some organisations in this state*.” (P_12_)

“*…we now teach them to stop FGM/C and also go on to tell them that the government will punish anybody reported to be doing it*, *as well as the parents of the girl or girls involved*.” (P_16_).

*Subtheme three*: *The continuation of FGM/C*. There was a division of the participants in terms of the continuation of FGM/C. However, the majority (11 of 17) said they did not support the continuation of this practice mostly because the government was against it. They explained:

“*I don’t do it*. *The government is against FGM/C*. *I don’t support FGM/C*. (P_12_)

“*No*, *it is dangerous and has many consequences as I have told you*.” (P_16_)

Some of the participants who were against the continuation of FGM/C supported the ‘new method.’ They explained they subjected their daughters to this procedure and even encouraged other women to practice this. Similarly, they were not willing to support the continuation of FGM/C openly. The participants said:

“*…I have tried all I could to persuade them to abandon the old types of FGM/C and embrace the new method…I did it for my baby girl…No*, *I only support the new method*.” (P_3_)

A few participants (five of 17) indicated strong support for the continuation of FGM/C. They supported the cutting method and gave reasons why they have continued to perform it for different families.

“*In my opinion*, *I love FGM/C as it reduces sexual urge…I don’t believe FGM/C causes difficult labour as big babies can equally cause difficult labour*.” (P_6_)

“*I think FGM/C prevents promiscuity in women*. *It also stops scratching of genitals in girls*” (P_5_)

“*Female adolescents can decide to get FGM/C*. *But mostly*, *I circumcise babies 8 or 9 days after birth at which time the cord has fallen…I don’t support the new method as it hurts the baby for long*. *It takes between 3 to 6 months to complete the procedure*. *It is better to do the circumcision once… I cut off the clitoris mainly and sometimes cut also little labia… No*, *I circumcise females; I can’t stay with an uncircumcised female…The clients don’t come to the hospital to have FGM/C*. *We go to their houses to circumcise them*.” (P_7_)

In summary, the participants reported that FGM/C was part of their culture and most of the females who presented at the health facilities had undergone circumcision. They explained they had stopped conducting FGM/C since the government placed a ban on the procedure, while some affirmed they still conducted the procedure for community members who requested it. Some participants compared FGM/C to male circumcision, while some encouraged the females to practice only the “new method,” not knowing it was a type of FGM/C. Factors such as ignorance and belief of various myths regarding FGM/C promoted the continuation of the practice.

## Discussion

This study focuses on an under-researched area and seems to be the first in providing a qualitative descriptive summary of the experiences of healthcare workers who care for genitally mutilated females in Africa [1]. The findings revealed the exposure of females to several complications due to their FGM/C, and this posed enormous challenges to the participants–healthcare workers. The complications due to FGM/C included a high risk for morbidity and mortality during childbirth, as it increased the risk for intrapartum haemorrhage, PPH, prolonged labour, infections, VVF and pain during and after childbirth. Available literature reports that FGM/C has a direct impact on maternal and neonatal mortality, as circumcised women have a 70% higher chance of PPH and twice the chance of death from childbirth complications compared to those who are not circumcised [36].

According to the participants, it was almost a norm to perform an episiotomy and prepare for a time-consuming suturing of the lacerations while assisting women during childbirth. Episiotomy was also reported to be effective in preventing severe lacerations during birth and this concurs with the report of Rodriguez et al. [37]. However, it was surprising that some of VVHWs and CHEWs reported that they also performed episiotomy while assisting the women during childbirth. This finding raises a concern for knowledge, skill and practice because, according to Aluko et al. [26], VVHWs and CHEWs were not certified to perform episiotomy in Nigeria.

Our study provided evidence that many of the female circumcisers in these communities were unskilled, with some removing so much of the external genitalia that the healthcare workers found it hard to identify the different anatomical parts. This complicated rendering healthcare services to the women during and after childbirth, and with suturing. The participants also experienced child mortality due to uncontrollable haemorrhaging following FGM/C, which, in addition to the other factors mentioned, emphasised the urgency of the call by UNICEF and UNFPA [3] for approaches that would bring a faster eradication of FGM/C.

It was evident that most of the participants in this study were natives of the study setting or nearby communities, and were part of the lifestyle, beliefs and culture of these communities. This is similar to the report of Shabila et al. [13], that the healthcare workers who were their study participants were from FGM/C practicing communities in Iraq and some of them were genitally mutilated. Therefore, despite the enormous challenges due to the FGM/C associated complications the women had, some participants, including registered nurses, justified the practice of FGM/C. They explained it was an age-old tradition embedded in their culture, the major reason that has sustained the practice of FGM/C to date. This finding raises concern as health care workers, especially, registered nurses and midwives are expected to know better and so act as advocates against FGM/C.

According to UNFPA, UNICEF Nigeria, and WHO [7, 36], some practitioners of FGM/C simply claim FGM/C was part of their culture and adhered to the practice irrespective of any harm it might have caused them or their loved ones. Although the participants considered culture as the main reason for FGM/C, they mentioned other factors such as ignorance, myths, and being born by a genitally mutilated mother. Similar factors were also reported by the WHO [7], and previous studies on FGM/C in Nigeria and other African countries [38, 39] as motivators of FGM/C.

The study participants thought the government’s support for the abandonment of FGM/C, led to an increase in infant FGM/C, the “new method,” which is type IV FGM/C, and FGM/C continuing secretly. There are similar trends reported in the literature. For instance, a study conducted in SE Nigeria found an increase in infant FGM/C and the new FGM/C method being used [40]. Another study also reported an increase in infant FGM/C in Tanzania [41], while yet another study opined that if people are not convinced about stopping FGM/C, they will continue with the practice in secrecy [42].

The participants were FGM/C practitioners until their state governments began to threaten to prosecute anyone caught doing FGM/C. It seems medicalisation of FGM/C was the norm until the anti-FGM campaigns/warnings by the state governments. This agrees with the findings on the reality of the medicalisation of FGM/C in Nigeria [39, 43]. The governments of Imo and Ebonyi States came into power in 2011 and May 2015 respectively, and began to increase the awareness about ending FGM/C and also reducing the medicalisation of the practice [44]. However, there is no evidence of anyone being arrested for FGM/C in SE Nigeria [11].

Although most of the participants joined the government in its campaign against FGM/C by teaching the community women to stop FGM/C, some could not give any reason why FGM/C should be stopped, except for the government’s threats of punishment, while some participants said they encourage the community women to imbibe the “new method” of FGM/C, which does not involve cutting off any tissue. It was unclear whether the participants who indicated support for FGM/C had a proper understanding of the effects of this procedure and therefore were reluctant to end it. Indeed, the lack of interest of some health professionals in fighting against FGM/C had been one of the problems in the ending the practice [12]. Some of the participants even equated FGM/C to male circumcision; this finding concurs with the available literature that reported poor knowledge of FGM/C among healthcare workers [12, 13, 15, 20]. To address difficulties in delivering appropriate and contextualised care to women who have undergone FGM/C, it is necessary for healthcare workers to have a proper understanding of the practice and its consequences. Besides, the government’s rules, and not own convictions, was the driver for those opposed to the continuation of FGM/C. Therefore, it might be difficult for these participants to convince their families and community members to stop FGM/C.

Some participants boldly declared they still support FGM/C and do it for various families on request. It was important to note that the participants who declared their support and continuation with FGM/C mentioned they do not perform the cutting in health facilities anymore but visit the homes of the clients when invited. Some participants who declared no support for the continuation of the practice gave justification for the practice. They explained how FGM/C was not a problem, and that they had successfully circumcised their daughters too. This finding might be explained by the reports by from other studies that some healthcare workers continue to perform FGM/C for reasons such as social acceptance [10, 12, 20]. However, Shabila et al reported that health workers who participated in their study in Iraq did not support or indicate they performed FGM/C [13].

Furthermore, the advice by some of the study participants about providing alternative sources of income for the traditional circumcisers, improving understanding on the need to stop FGM/C and enforcing the anti-FGM/C law, could be good initiatives in the attempt to hasten the eradication of FGM/C.

### Limitations

The conducting of this study was in communities of two local government areas in two states of SE Nigeria, which reportedly had the highest prevalence of FGM/C in the area. The results therefore may not be generalised to all the communities in Nigeria. Although the researcher believes a rigorous process was followed in terms of the translation of the interviews, back-translation, which is the translation method used by many researchers [45, 46], was not followed.

## Conclusions

The participants experienced challenges in caring for the genitally mutilated females. The genitally mutilated women were at risk for morbidity and mortality during childbirth, as FGM/C increased the risk for intrapartum haemorrhage, PPH, prolonged labour, infections, VVF, and pain during and after childbirth. The participants felt ill-prepared to manage these complications as they lacked advanced skills, especially when assisting genitally mutilated women during childbirth. Irrespective of this complex situation, the participants justified FGM/C by explaining it was an age-old cultural practice, while some supported its continuation.

### Recommendations

Educational programmes involving all cadres of healthcare workers could prepare them to cope with the challenges they face when caring for genitally mutilated females. However, this will not prevent the complications of FGM/C they deal with. Eradicating FGM/C would be the only solution.

## Supporting information

S1 Table FGM/C interview guide for healthcare workers (English language). (DOCX)

S2 Table FGM/C interview guide for healthcare workers (Igbo language). (DOCX)

## References

[1] Obiora OL, Maree JE, Mafutha N (2019) Female Genital Mutilation in Africa: Scoping the Landscape of Evidence. International Journal of Africa Nursing Sciences 100189

[2] Onuoha C (2018) Female Genital Mutilation persists despite outlaw. Vanguard News Nigeria

[3] UNICEF, UNFPA (2018) A race against trends. In: UNICEF. https://www.unicef.org/media/media_102559.html. Accessed 12 Feb 2019

[4] United Nations Children’s Fund, Gupta GR(2013) Female Genital Mutilation/Cutting: A statistical overview and exploration of the dynamics of change. Reproductive Health Matters 184–190

[5] WHO F (2008) WMH. Eliminating female genital mutilation: an interagency statement. UNAIDS, UNDP, UNECA.

[6] Davis G, Jellins J (2019) Female genital mutilation: Obstetric outcomes in metropolitan Sydney. Australian and New Zealand journal of obstetrics and gynaecology 10.1111/ajo.12954 30734267

[7] WHO (2018) Female genital mutilation. https://www.who.int/news-room/fact-sheets/detail/female-genital-mutilation. Accessed 13 Feb 2019

[8] Berg R, Underland V (2014) Immediate health consequences of female genital mutilation/cutting (FGM/C). Knowledge Centre for the Health Services at The Norwegian Institute of … 29320014

[9] Berg RC, Denison E (2013) A tradition in transition: factors perpetuating and hindering the continuance of female genital mutilation/cutting (FGM/C) summarized in a systematic review. Health care for women international 34:837–859 10.1080/07399332.2012.721417 23489149 PMC3783896

[10] Morhason-Bello IO, Fagbamigbe AF, Kareem YO, Ojengbede OA (2020) Economic status, a salient motivator for medicalisation of FGM in sub-Saharan Africa: Myth or reality from 13 national demographic health surveys. SSM—Population Health 11:100602 10.1016/j.ssmph.2020.100602 32478164 PMC7251377

[11] 28 Too Many (2018) The law and FGM: An overview of 28 African Countries. In: 28 Too Many. https://www.28toomany.org/static/media/uploads/Law%20Reports/the_law_and_fgm_v1_(september_2018).pdf. Accessed 24 Jul 2019

[12] Reig‐Alcaraz M, Siles‐González J, Solano‐Ruiz C (2016) A mixed-method synthesis of knowledge, experiences and attitudes of health professionals to Female Genital Mutilation. Journal of Advanced Nursing 72:245–260 10.1111/jan.12823 26434563

[13] Shabila NP, Ahmed HM, Safari K (2017) Knowledge, attitude, and experience of health professionals of female genital mutilation (FGM): A qualitative study in Iraqi Kurdistan Region. Health Care for Women International 38:1202–1218 10.1080/07399332.2017.1365867 28841367

[14] Utz-Billing I, Kentenich H (2008) Female genital mutilation: an injury, physical and mental harm. Journal of Psychosomatic Obstetrics & Gynecology 29:225–229 19065392 10.1080/01674820802547087

[15] Leye E, Van Eekert N, Shamu S, Esho T, Barrett H, ANSER (2019) Debating medicalization of Female Genital Mutilation/Cutting (FGM/C): learning from (policy) experiences across countries. Reprod Health 16:158 10.1186/s12978-019-0817-3 31675972 PMC6823951

[16] Rafferty Y (2013) International dimensions of discrimination and violence against girls: A human rights perspective. Journal of International Women’s Studies 14:1–23

[17] Yoder PS, Abderrahim N, Zhuzhuni A (2004) Female genital cutting in the Demographic and Health Surveys: a critical and comparative analysis.

[18] Cappa C, Moneti F, Wardlaw T, Bissell S (2013) Elimination of female genital mutilation/cutting. The Lancet 382:1080–1081 10.1016/S0140-6736(13)61532-9 23886657

[19] Berggren V, Salam GA, Bergström S, Johansson E, Edberg A-K (2004) An explorative study of Sudanese midwives’ motives, perceptions and experiences of re-infibulation after birth. Midwifery 20:299–311 10.1016/j.midw.2004.05.001 15571879

[20] Obianwu O, Adetunji A, Dirisu O (2018) Understanding medicalisation of female genital mutilation/cutting (FGM/C): A qualitative study of parents and health workers in Nigeria. 10.31899/rh6.1039

[21] Christoffersen‐Deb A (2005) “Taming tradition”: medicalized female genital practices in western Kenya. Medical anthropology quarterly 19:402–418 10.1525/maq.2005.19.4.402 16435647

[22] World Health Organization (2010) WHO | Global strategy to stop health-care providers from performing female genital mutilation. In: WHO. https://www.who.int/reproductivehealth/publications/fgm/rhr_10_9/en/. Accessed 29 Jul 2020

[23] Budiharsana M, Amaliah L, Utomo B (2003) Female circumcision in Indonesia. Extent, implications and possible interventions to uphold women’s health rights. 10.31899/rh2.1005

[24] UNFPA, UNICEF Nigeria (2016) Real people, real stories. Join us to enf FGM in Nigeria! https://nigeria.unfpa.org/sites/default/files/pub-pdf/FGM%20for%20web%20human%20stories.pdf. Accessed 30 Mar 2019

[25] NPC [Nigeria], ICF International (2014) Nigeria Demographic and Health Survey 2013. https://dhsprogram.com/pubs/pdf/FR293/FR293.pdf. Accessed 24 Feb 2019

[26] Aluko JO, Anthea R, Marie Modeste RR (2019) Manpower capacity and reasons for staff shortage in primary health care maternity centres in Nigeria: a mixed-methods study. BMC Health Serv Res 19:10 10.1186/s12913-018-3819-x 30616598 PMC6322346

[27] 28 Too Many (2018) Nigeria: The Law and FGM. In: nigeria_law_report_v1_(june_2018).pdf. https://www.28toomany.org/static/media/uploads/Law%20Reports/nigeria_law_report_v1_(june_2018).pdf. Accessed 15 Apr 2020

[28] Sandelowski M (2000) Whatever happened to qualitative description? Research in nursing & health 23:334–340 10.1002/1098-240x(200008)23:4&lt;334::aid-nur9&gt;3.0.co;2-g 10940958

[29] Etikan I, Musa SA, Alkassim RS (2016) Comparison of convenience sampling and purposive sampling. American journal of theoretical and applied statistics 5:1–4

[30] Leech BL (2002) Asking Questions: Techniques for Semistructured Interviews. Political Science & Politics 35:665–668

[31] Bradford A (2017) Deductive Reasoning vs. Inductive Reasoning. https://www.livescience.com/21569-deduction-vs-induction.html. Accessed 24 May 2019

[32] Elo S, Kyngäs H (2008) The qualitative content analysis process. Journal of advanced nursing 62:107–115 10.1111/j.1365-2648.2007.04569.x 18352969

[33] Malterud K (2001) Qualitative research: standards, challenges, and guidelines. The lancet 358:483–488 10.1016/S0140-6736(01)05627-6 11513933

[34] Pillow W (2003) Confession, catharsis, or cure? Rethinking the uses of reflexivity as methodological power in qualitative research. International journal of qualitative studies in education 16:175–196

[35] Booth A, Hannes K, Harden A, Noyes J, Harris J, Tong A (2014) COREQ (consolidated criteria for reporting qualitative studies). Guidelines for reporting health research: A user’s manual 214–26

[36] UNFPA, UNICEF Nigeria (2016) Real people, real stories. Join us to enf FGM in Nigeria! https://nigeria.unfpa.org/sites/default/files/pub-pdf/FGM%20for%20web%20human%20stories.pdf. Accessed 1 May 2019

[37] Rodriguez MI, Seuc A, Say L, Hindin M (2016) Episiotomy and obstetric outcomes among women living with type 3 female genital mutilation: a secondary analysis. Reproductive Health. 10.1186/s12978-016-0242-9 27724946 PMC5057400

[38] (2019) Nigeria28 Too Many. https://www.28toomany.org/country/nigeria/. Accessed 24 Feb 2019

[39] Okeke T, Anyaehie U, Ezenyeaku C (2012) An overview of female genital mutilation in Nigeria. Annals of medical and health sciences research 2:70–73 10.4103/2141-9248.96942 23209995 PMC3507121

[40] Onyima BN (2015) Infant-female genital mutilation (IFGM) in cities and the role of women in perpetuating FGM: a probe on why FGM persists in urban centers in Nigeria. Mgbakoigba: Journal of African Studies 4:1–12

[41] Galukande M, Kamara J, Ndabwire V, Leistey E, Valla C, Luboga S (2015) Eradicating female genital mutilation and cutting in Tanzania: an observational study. BMC public health 15:1147 10.1186/s12889-015-2439-1 26584655 PMC4653879

[42] Getachew I (2006) Battling an ancient tradition: Female genital mutilation in Ethiopia. In: UNICEF. https://www.unicef.org/protection/ethiopia_34881.html. Accessed 23 Apr 2019

[43] Eze M (2017) Groups tackle female genital mutilation in Imo, Ebonyi. The Sun Nigeria

[44] Cahoon B (nd) Nigerian States. http://www.worldstatesmen.org/Nigeria_federal_states.htm#Imo. Accessed 2 May 2019

[45] Maneesriwongul W, Dixon JK (2004) Instrument translation process: a methods review. Journal of Advanced Nursing. 10.1111/j.1365-2648.2004.03185.x 15369498

[46] Chen H-Y, Boore JR (2010) Translation and back-translation in qualitative nursing research: methodological review. Journal of Clinical Nursing 19:234–239 10.1111/j.1365-2702.2009.02896.x 19886874

