# Supplementary material for: “A lot of them have scary tears during childbirth…” experiences of healthcare workers who care for genitally mutilated females
Source: PLoS One. 2021 Jan 29;16(1):e0246130. doi: 10.1371/journal.pone.0246130 (PMC7845945; doi:10.1371/journal.pone.0246130)
Supplement: S1 Table — (DOCX) [file pone.0246130.s001.docx]

**Interview guide**

**“A lot of them have scary tears during childbirth…” Experiences of Healthcare Workers who Care for Genitally Mutilated Females**

**English language version**

Section A: General Information

| Nos | Categories | Findings |
| --- | --- | --- |
| 1. | Age |  |
| 2. | Cultural group |  |
| 3. | Religion |  |
| 4. | Marital Status |  |
| 5. | Highest Educational level |  |
| 6 | Years of practice in this community |  |
| 7. | Years of residency in the community |  |
| 8. | Have you witnessed female circumcision? |  |
| 9. | If yes to question 8 above, how many times? |  |
| 10 | Age at which females are circumcised |  |
| 11 | Consent obtained |  |
| 12 | What type (s) of female circumcision is done in this community? |  |
| 13 | Support for FGM/C |  |

Section B: Main Question

*Please tell me your experience of caring for genitally mutilated females in this community*
