# Supplementary material for: “A lot of them have scary tears during childbirth…” experiences of healthcare workers who care for genitally mutilated females
Source: PLoS One. 2021 Jan 29;16(1):e0246130. doi: 10.1371/journal.pone.0246130 (PMC7845945; doi:10.1371/journal.pone.0246130)
Supplement: S2 Table — (DOCX) [file pone.0246130.s002.docx]

**Interview guide**

**“A lot of them have scary tears during childbirth…” Experiences of Healthcare Workers who Care for Genitally Mutilated Females**

**Igbo language version**

Nkebi nke mbu: ozi izugbe

| Nos | Categories | Findings |
| --- | --- | --- |
| 1. | Afọ ole ka ịdị? |  |
| 2. | Otu omenaala |  |
| 3. | Okpukpe |  |
| 4. | Ọnọdụ alụmdi na nwunye |  |
| 5. | kedu nzere agumakwukwo kachasi elu inwere? |  |
| 6 | Afọ ole ka ị rụgoro ọrụ na obodo a? |  |
| 7. | Afọ ole ka ị bigoro na obodo a? |  |
| 8. | I hutugo ebe a n’ebi nwanyi úgwù? |  |
| 9. | Oburu n’usa gi buru ee nye ajụjụ nke 8 di n’elu, ugboro ole? |  |
| 10 | Afọ ole ka nwanyị ga-adi tutu ebie ya úgwù? |  |
| 11 | Ana-enweta nkwado site n’ aka umunwanyị tutu ebi e ha úgwù? |  |
| 12 | Olee ụdị úgwù a na-ebi n'obodo a? |  |
| 13 | I kwadoro omenaala ibi nwanyi úgwù? |  |

Nkebi nke abuọ: isi ajụjụ

*Biko gwa m ahumihe gi banyere ilekota ahu ike um nwanyi ebiri* úgwù *na obodo a*
